# Prevalence of undernutrition and its associated factors among pregnant women in Konso district, southern Ethiopia: a community-based cross-sectional study

**DOI:** 10.1186/s40795-021-00437-z

**Published:** 2021-07-12

**Authors:** Deyganto Gergito Gelebo, Mathewos Alemu Gebremichael, Gistane Ayele Asale, Dessalegn Ajema Berbada

**Affiliations:** 1 Save the Children International, Konso Project Office, Konso, Ethiopia; 2 School of Public Health, College of Medicine and Health Sciences, Arba Minch University, Arba Minch, Ethiopia

**Keywords:** Undernutrition, Maternal malnutrition, Pregnant women, Konso, Ethiopia

## Abstract

**Background:**

Undernutrition during pregnancy is an important public health problem. It is highly prevalent in Ethiopia but not sufficiently addressed yet. Hence, this study aimed to assess the prevalence of undernutrition and its associated factors among pregnant mothers in Konso district, Ethiopia.

**Methods:**

Community-based cross-sectional study was conducted with a qualitative inquiry from December 2018 to January 2019. A multistage stratified sampling technique was used to select 527 subjects and quantitative data were collected from these subjects using a structured interviewer-administered questionnaire and additionally qualitative data were generated through two focus group discussions among purposely selected discussants. Mid upper arm circumference (MUAC) was measured by standard non-stretchable MUAC tape. Data were entered into Epi-data version3.1 and analyzed by SPSS version 21. In multivariable binary logistic regression, a statistically significant association was declared at *p*-value < 0.05 while thematic framework analysis was employed for the qualitative data.

**Results:**

Among 501 participants, the overall prevalence of undernutrition (MUAC < 23 cm) was 43.1% (95% CI 38.7–47.5%). Household food security (AOR = 3.1; 95% CI: 2.1–4.6), low dietary diversity score (AOR = 4.9; 95% CI: 2.6–9.2), medium dietary diversity score (AOR = 2.3; 95% CI: 1.2–4.7), absence of latrine (AOR = 1.8; 95% CI: 1.2–2.6) and having family resource decision making by husband only (AOR = 1.7; 95% CI: 1.1–2.6) were significantly associated factors. Traditional believes to restrict food such as egg, milk, and milk products, avocado for women, weak nutrition education and malnutrition screening program, daily consumption of locally prepared alcoholic drink called “Cheka”, drought-prone nature of this setting, traditional way of farming practices and low socio-economic status were found to be barriers for women’s undernutrition.

**Conclusions:**

The prevalence of undernutrition was found to be higher than previously reported findings. Household food security, dietary diversity, latrine availability, family resource decision making, food restrictions, weak maternal nutrition education, and malnutrition screening program, the practice of depending on a local alcoholic drink called “Cheka”, drought, traditional way of farming and low socio-economic status were identified factors. Hence, interventions targeting maternal nutrition education, hygiene, and sanitation promotion, household food insecurity improvement strategies should be implemented to improve the nutritional status of pregnant women.

## Background

Undernutrition is an important public health issue particularly for vulnerable groups including children and women of childbearing age especially pregnant mothers [1]. Undernutrition is a serious global health problem. About 795 million people are undernourished mostly in low and middle-income countries and the problem is most critical during pregnancy [2]. Globally, undernutrition is contributing to the deaths of 3.5 million mothers and under 5 years of age children each year. It is estimated that 13 million children are annually born with IUGR (intrauterine growth retardation), 112 million are underweight secondary to undernutrition during pregnancy [3, 4].

Pregnancy is a critical phase in which mothers need optimal nutrients of good qualities of food to support the developing fetus [5]. An adequate supply of nutrients and oxygen for the mother to her fetus is one of the factors that are critical for fetal survival. The ability of the mother to provide nutrients for her baby depends upon the nutritional status, body size, and body composition of the mother and all of which are being established throughout the life of her fetus [6].

Undernutrition in pregnant mothers is a key contributor to many problems. It makes the women more susceptible to diseases, more risk of having miscarriages, poor fetal growth, low birth weight, infant morbidity, and mortality [7]. It can also cause long-term irreversible and detrimental cognitive-motor and health impairment [7]. In addition to serious consequences of health, the economy can be also affected by undernutrition. The high prevalence of undernutrition can hampers economic growth and preserves poverty both directly, through a loss of productivity due to poor physical condition, and indirectly, through poor cognitive function and learning deficits [8, 9].

Evidence showed that the burden of undernutrition among pregnant women is high. The prevalence of pregnant women’s undernutrition in India 5%, China 21%, Sri Lanka 15%, Nigeria 10–40% [10–12]. In Ethiopia, the prevalence of undernutrition in pregnant women varies from 9.2–41.2%. It was reported in Central Refit Valley, Wondogenet District in Ethiopia, Silte zone, Southern Ethiopia, Dessie Northern Ethiopia, Eastern Ethiopia, Gondar town and hospital in Northern Ethiopia, humanitarian setting of Ethiopia, Shashemene, in a rural community in southern Ethiopia, 31.7, 9.2, 21.8, 19.8%, 19.5, 16.2, 14.4, 24, 34, 35.5, and 41.2%, respectively [13–22]. The most acceptable explanation for this wide variation is likely to be the fact that variation in the contextual factors of pregnant women’s undernutrition.

Previous studies identified different risk factors for pregnant women undernutrition. These factors were: age, educational status, family size, marital status, social class, the height of the mother, household food insecurity, household asset, homeownership, household saving, financial constraint, ownership of life stock, job loss, and low level of income, gender of household head, cultivated farmland size, inadequate dietary intake, women’s knowledge about nutrition, residence, iron supplementation during pregnancy, meal frequency per day, trimester, perceptions imposing dietary restriction on certain food, health status of pregnant mothers, antenatal care service dissatisfaction, and family planning utilization before current pregnancy [23–34].

Due to differences in characteristics of socio-economic, culture, ethnicity, and geographical location, associated factors for undernutrition in pregnant women might not be the same across different regions of Ethiopia. The government of Ethiopia launches a national nutrition program. Maternal nutrition is one of its targets that needs updated data which is essential to develop effective intervention strategies to prevent undernutrition of pregnant women and children. In addition to this, there is limited data on prevalence and factors associated with maternal undernutrition particularly in the southern part of Ethiopia. According to the 2010 E. C Konso district health office report, the prevalence of undernutrition among pregnant mothers was more than 30% [35], but the risk factors were not well understood. And also the cultural practices which restrict some important foods during pregnancy are commonly practiced in Konso. Therefore, the undernutrition of pregnant women needs to be assessed in a specific context to develop effective interventions. Hence, this study determined the prevalence of undernutrition and its associated factors among pregnant women in Konso district, Southern Ethiopia.

## Methods

### Study area, study design, and study period

The community-based cross-sectional study design with focus group discussion (FGD) was conducted in Konso District from 1st December 2018 to 31st January 2019. Konso District is located 595 Kilometers from Addis Ababa (capital city of Ethiopia) and 365 km from Hawassa (the capital city of the south region). Based on the Konso district administrative population profile the district has a total population of 270,837 from this 132,710 male and female 138,127. The District has 50 health posts, 11 health centers, and 1 primary hospital, and 25 private clinics which are providing health services for the community.

### Population

The source population of this study was all pregnant women in Konso District. The study population was all pregnant women in the selected Kebeles (small administrative unit in Ethiopia) of Konso District. All pregnant women who were residents of the Konso district for at least 6 months or above were included in the study whereas those pregnant women who were seriously ill, those who had hand deformity were excluded.

### Sample size determination and sampling technique

The sample size was computed using single population proportion formula; considering 95% confidence level, 5% margin of error, 1.5 design effect, 31.8% hypothesized prevalence of undernutrition among pregnant women which has been taken from a study conducted in the central rift valley of Ethiopia [15] and by adding 5% none response rate the final sample size for this study was 527. A multi-stage stratified sampling which was followed by a systematic sampling technique was applied to reach each study participant. The kebeles in the district were stratified into rural and urban. One urban and eight rural Kebeles were selected by using the lottery method from a total of forty-one kebeles in the Konso district. Then by using health extension workers housing registration, the total number of households with pregnant women (2277) was accessed and the sampling interval was calculated. Finally, households with eligible pregnant women were selected using a systematic random sampling technique. For those households with more than one pregnant woman, one pregnant woman was selected by using the lottery method. Data collators visited the house on the next day when the pregnant women were not available at home and the pregnant women who were not available during the second visit were recorded as non-response, and then nearby household was considered.

### Qualitative sampling procedure

Two FGD were conducted with 12 subjects in each group who were purposively selected. A situational analysis was done before conducting the focus group discussion (FGD) to minimize errors in the selection of participants. The key informants were a case team leader for maternal health and a supervisor assigned for each Kebele and agricultural expert (agriculture and natural resource head). In each FGD pregnant women, their husbands, and health extension workers participated. To minimize the possible bias in a selection of the study participants, we made sure to emphasize that we want a group of people that can express a range of views, to be able to have a proper discussion. A smooth discussion environment was created and we tried to encourage communication and interaction during the focus group discussion in every possible way. FGDs were held in a neutral setting which encourages participants to express their views freely. We made sure that there were no disturbances, adequate lighting, and ventilation as it was the hottest season during the data collection period, and also there were cold beverages including water. Materials that were necessary to conduct the focus group discussion (FGD) were prepared before a discussion like the FGD guideline, voice recorders, notebooks, pen, and pencils. To create a conducive discussion area, the chairs were arranged in a circle.

### Study variables

The dependent variable in this study was undernutrition among pregnant women. The independent variables were: Socio-demographic factors:-age, marital status, husband education maternal education, family size, polygamy. Maternal related factor: - parity, family planning utilization before current pregnancy, birth interval, receiving iron supplementation, ANC follows up, ANC satisfaction, Nutrition knowledge, Illness, History of abortion, History of stillbirth, dietary diversification (24-h recall), meal frequency, Socio-Cultural factors: -food taboo and food restriction during pregnancy, decision making on household assets, family stable food. Economic factors:-Family source of food, farmland ownership, employment (maternal& husband job) status, household income, wealth index. Hygiene and sanitation-related factors:-access to water and sanitation facilities, such as latrine availability & utilization, family source of water, distance to get water. Food Security-related factors - food accessibility and availability.

### Data collection tool and procedure

An interviewer-administered structured questionnaire was used to collect the data for the quantitative part of the study and qualitative data was collected using two focus group discussions (FGDs). The tool included: Socio-demographic factors adapted from the Ethiopian demographic and health survey (EDHS 2016) [36], Maternal related factor, Socio-Cultural factors, Economic factors, Hygiene and Sanitation related factors, and food Security related factors.

To determine the nutritional status of pregnant mothers, MUAC (mid-upper arm circumference) was used. Mid upper arm circumference of the left arm was measured triplicate using a non-stretchable standard MUAC tape to the nearest 0.1 cm with no clothing on the arm. The mean of triplicate measurement was taken. The value of MUAC below 23 cm was considered as undernourished and MUAC ≥23 cm was considered as normal nutritional status [34, 37].

Dietary diversity information of individual respondents was collected using the 24-h recall method and women dietary diversity score model questionaries’ of nine food groups with food listing method in which list of food items replaced by common foods in local context included in the questioner [38].

Household food security was measured by the household food insecurity access scale (HFIAS) which is an adopted approach in estimating the prevalence of food insecurity in the united states (USA) and was used to estimate the food insecurity among study participants [39]. HFIAS prevalence indicator categorizes households into four levels of food security as food secure, mildly, moderately, and severely food insecure [25]. HFIAS yes or no questions were used to collect information on the food security status of the household followed by the occurrence of the situation if the response is yes [39]. For the occurrence of once or twice, it was recorded as rarely if the occurrence is 3–10 times it was categorized as sometimes and if the occurrence was more than ten times in the past 4 weeks it was categorized as often [26, 40].

Purposively selected subjects participated in the two focused group discussions for qualitative data. The composition of focus group discussion participants was, pregnant mothers, elders or mother’s in-low, and their husbands, health extension workers were engaged in the discussion separately to facilitate the expression of opinions without fear and a key informant interview was held with the health department head and agricultural expert (agriculture and natural resource head). The discussion was conducted at community meeting places, and the information was collected using open-ended questions. Note-taking and tape recording were used to document the appropriate information and detect redundant responses. Identified redundant responses were considered to be saturated and removed every evening after triangulation the day work during preliminary analysis.

### Data quality assurance

To assure data quality, training was given to data collectors and supervisors before data collection. The data collection tool was pre-tested in 5% of the sample size. The pretest was conducted on individuals having similar characteristics of the study in Kebele which was not selected in this study. After the pre-test, the instrument was modified accordingly. Supervisors supervised the data collection process and checked the completeness of the questionnaire daily. Besides, principal investigators carefully cleaned the data and entered collected data into computer software.

### Data processing and analysis

Epi-data version 3.1 statistical software was used for data entry and exported to SPSS version 21 for analysis. Descriptive statistics like mean, standard deviation, frequencies, and percentages were computed. Bivariable and multivariable logistic regression was used to determine the degree of association between independent and dependent variables. All variables with a *p*-value less than 0.2 in the bivariable analysis were entered into a multivariable logistic regression. The presence of an association between dependent and independent variables was checked with an adjusted odds ratio with 95% confidence intervals. Then the statistical significance was declared at a *p*-value less than 0.05 and adjusted odds ratio interval which excluded one. Assumption of logistic regression such as; meaningful coding, multicollinearity, and outliers checking was done before logistic regression model analysis. Multicollinearity was also checked by using Variance inflation factors and Tolerance test. The Hosmer-Lemeshow tests were checked to assess the goodness-of-fit model and it was a good fit(*p*-value > 0.05). The wealth index of individual respondent families was also analyzed by using principal component analysis.

## Results

### Socio-demographic characteristics of the respondents

From the total of 527 pregnant women, 501 participated in this study making the response rate 95%. The mean age of the respondents was 28.4 ± 5.3 years, range between 18 and 45 years, and the majority of the study subjects were age between 25 and 34 years. Regarding the marital status, 486(97%) of the study participants were married and living with their husbands and the rest were unmarried. Among the total, 92(18.4%) of the pregnant women had husbands who have another wife or polygamous. The average family size of the study subjects was 5.9 ± 2.8, with a maximum family size of 14 and a minimum of 2. The majority 335 (66.8%) of the study participants were Protestants religious followers, 95(18.9%) were Orthodox and the rest were traditional believers. Regarding educational status, 341(68.1%) were illiterate, 54(10.8%) were can read and write, 68(13.6%) were primary, and 38(7.6%) were secondary and above educational status (Table 1).

**Table 1:** Sociodemographic characteristics of respondents in Konso district, southern Ethiopia 2019 (*n* = 501)

Variable	Category	Frequency	Percent (%)
Marital status	Married	486	97.0
	Unmarried	15	3.0
Age of women in years	18–24	103	20.5
	25–34	323	64.5
	35–45	75	15.0
Family size in number	2–5	249	49.7
	6–8	170	33.9
	≥ 9	82	16.4
Religion	Protestant	335	66.9
	Orthodox	95	19.0
	Cultural believers	71	14.2
Women’s educational status	Illiterate	341	68.1
	Can read and write	54	10.8
	Primary education	68	13.6
	Secondary and Above	38	7.6
Husband educational status	Illiterate	273	54.5
	Can read and write	67	13.4
	Primary education	61	12.2
	Secondary and Above	100	20.0
Women’s occupational status	Farmer	397	79.2
	Merchant	42	8.4
	Daily worker	40	8.0
	Government worker	22	4.4
Husband’s occupational status	Farmer	359	71.7
	Merchant	44	8.8
	Daily worker	44	8.8
	Government worker	54	10.8
Husbands with two or more wives.	Yes	92	18.4
	No	409	81.6
Average family income (Ethiopian birr)	< 500	350	69.9
	500–1000	63	12.5
	1001–1500	23	4.6
	> = 1501	65	12.9
Decision-making power on households asset	Husband only	183	36.5
	Wife only	14	2.7
	Husband and wife	304	60.6
Wealth index	Poor	392	78.2
	Medium	67	13.4
	Rich	29	5.8
	Very rich	13	2.6

### Food security status

**Fig. 1:**
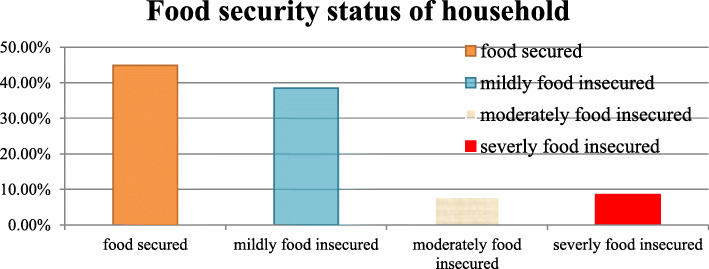
Household food security Status of respondents in Konso district, southern Ethiopia, 2019

Regarding the food security status of the household of the study subjects, about 244(44.7%) were food secured families but the rest are food insecure (Fig. 1).

### Maternal related characteristics of respondents

A total of 359 (71.6%) of mothers received pregnancy-related nutrition information from health professionals and health extension workers. Regarding meal frequency, 42(8.4%) reported eating once a day, 302(60.2%) of them had reported eating three times and more a day. The majority of 432(86.3%) of the study participants were multigravida whereas only 69(13.7%) were primigravida. Regarding gestational age 46(9.2%) were in the first trimester and 304(60.7%) of pregnant mothers were in third trimesters of gestational age.

From total undernutrition cases, more than 50 % of mothers or 134(62.0%) are reported from mothers in third trimester gestational age. Regarding the birth interval, 141(28.1%) of mothers reported that the interval between children is below 24 months and the rest was above 24 months or more. A total of 336(67.1%) women reported that they used the modern family planning method before their current pregnancy and 105(20.9%) of women reported that the current pregnancy was not intended. A total of 34(6.8%) had a history of stillbirth and the rest 467(93.2%) did not experience stillbirth. Those mothers had a history of abortion were 41(8.2%) and the rest had never experienced abortion. Regarding ANC attendance majority of 339(67.7%) of women attended antenatal clinic twice or more and 61(12.2%) of pregnant mothers never attended ANC clinic (Table 2).

**Table 2:** Maternal related characteristics of respondents in Konso district, southern Ethiopia 2019 (*n* = 501)

Variable	Category	Undernutrition		Total
		Yes	No	
Number of pregnancy	1	29 (5.8%)	40 (8.0%)	69 (13.8%)
	2–5	140 (27.9%)	186 (37.1%)	326 (65.1%)
	≥ 6	47 (9.4)	59((11.8%)	106 (21.2%)
Historyof family planning utilization	Used	70 (14.0%)	95 (19.0)%	165 (32.9%)
	Not used	146 (29.1%)	190 (37.9%)	336 (67.1%)
Received nutrition information	Yes	154 (30.7%)	205 (40.9%)	359 (71.7%)
	No	62 (12.4%)	80 (16.0%)	142 (28.3%)
Gestational age	1st trimester	19 (3.7%)	40 (8.0%)	69 (13.8)
	2nd trimester	63 (12.6%)	186 (37.1%)	326 (65.1%)
	3rd trimester	134 (26.7%)	59((11.8%)	106 (21.2%)
Knowledge of additional meal during pregnancy	Yes	170 (33.9%)	225 (44.9%)	395 (78.8%)
	No	46 (9.2%)	60 (11.9)	106 (21.2%)
Antenatal attendance	Yes	244 (48.7%)	180 (35.9%)	424 (84.6%)
	No	41 (8.2%)	36 (7.2%)	7715.4%)
History of still birth	Yes	14 (2.8%)	20 (4%)	34 (6.8%)
	No	270 (53.9%)	197 (39.3%)	467 (93.2%)
Received iron during ANC	Yes	225 (44.9%(	170 (33.9%)	395 (78.8%)
	No	60 (12%)	46 (9.2%)	106 (21%)

### Dietary diversity score

**Fig. 2:**
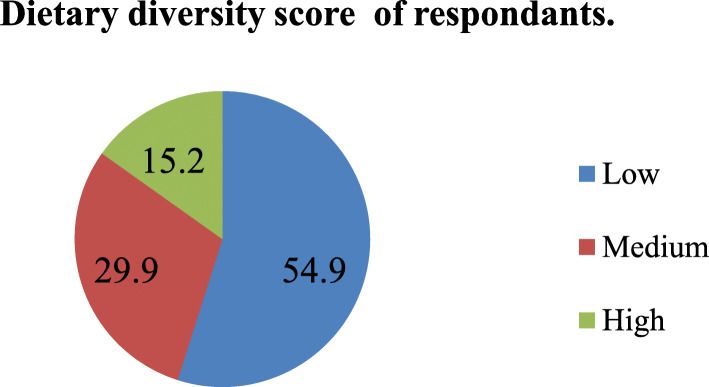
Dietary diversity score of the respondents in Konso district, southern Ethiopia, 2019

According to the dietary diversity assessment result, the majority (54.9%) of women scored low dietary diversity score or DDS and the rest met the standard dietary diversity score criteria of medium and high (Fig. 2). From food groups consumed in the previous 24 h, a total of 399 (79.7%) consumed starch staples (grains, white roots and tuber, and plantains), 185 (36.9%) pulses (beans, peas, and lentils), 275 (54.9%) nuts and seeds, 85 (16.9%) dairy, 76(15.2%) meat, poultry, and fish, 21(0.4%) eggs, 83 (16.6%) dark green leafy vegetables, 79(15.8%) other vitamin A-rich fruits and vegetables, 2028 (45.5%) other vegetables and 195 (38.9%) other fruits.

### WASH-related characteristics of study participants

**Fig. 3:**
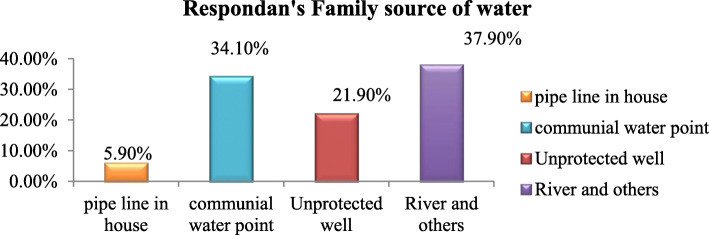
Household source of water for respondents in Konso district, southern Ethiopia, 2019

According to the water, hygiene, and sanitation status of the study subjects, 230(45.9%) households did not have pit latrine and the latrine coverage was only 54.1%. Regarding time taken to fetch water, 134(26.7%) of mothers were traveling 1 h and more to get water and 188(37.5%) mothers were traveling less than 30 min to get water. The majority 190 (37.9%) of respondents was using the river as a source of water followed by communal water point (Table 3, Fig. 3).

**Table 3:** WASH-related Factor of respondents in Konso district, southern Ethiopia, 2019 (*n* = 501)

Variable	Category	Undernutrition		Total
		Yes	No	
Place of defecation	Bush	103 (20.6%)	101 (20.15%)	204 (40.7%)
	Community pit	16 (3.2%)	10 (2.0%)	26 (5.2%)
	Use their own latrine	97 (19.4%)	174 (34.7%)	271((54.1%)
Latrine availability	Yes	99 (19.8%)	172 (34.3%)	271 (54.1%)
	No	117 (23.4%)	113 (22.6%)	230 (45.9%)
Time to fetch water	< 30 min	80 (15.9%)	108 (21.6%)	188 (37.5%)
	30–60 min	76 (15.2%)	103 (20.6%)	179 (35.7%)
	≥ 60 min	60 (11.9%)	74 (14.8%)	134 (26.7%)

### Prevalence of undernutrition among the study participants

**Fig. 4:**
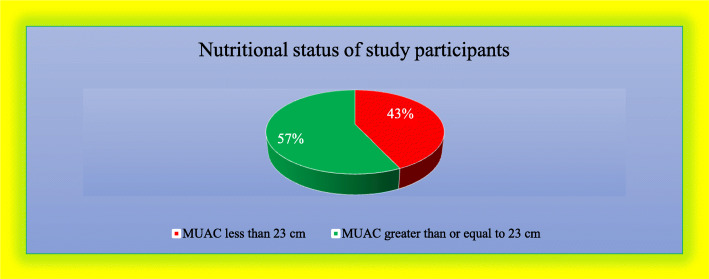
Nutritional status of respondents in Konso district, southern Ethiopia, 2019

The result of this study found, 216(43.1%) (95% CI: 38.8%-47.5) of the pregnant women were undernourished (MUAC < 23 cm) and those with MUAC greater than or equal to 23 cm were 285(56.9%) (Fig. 4). The mean MUAC was 22.9 ± 1.4 (SD), the minimum and maximum MUAC for the study subject was 18 cm and 26 cm respectively.

### Factors associated with undernutrition

Both Bivariable and multivariable logistic regression analyses were employed. In the bivariable analysis variables such as religion, marital status, mothers occupational status, husbands occupational status, average family income, source of water, resource decision making power on the household asset, household food security status, history of stillbirth, knowledge of additional meal during pregnancy, latrine availability, dietary diversity score, and iron supplementation was significantly associated with undernutrition. Variables with a *p*-value of < 0.25 in the bivariable logistic regression analysis were entered into multivariable logistic regression analysis.

In multivariable analysis, household food insecurity (AOR = 3.1; 95%CI: 2.1–4.6), low dietary diversity score (AOR = 4.9; 95%CI: 2.6–9.2), medium dietary diversity score (AOR = 2.3; 95%CI: 1.2–4.7), absence of household latrine (AOR = 1.8; 95%CI: 1.2–2.6) and having family resource decision making by husband only (AOR = 1.7; 95%CI: 1.1–2.6) were significantly associated with undernutrition (Table 4).

**Table 4:** Factors associated with nutritional status of pregnant women in Konso district, Southern Ethiopia, 2019 (*n* = 501)

Variables	Category	Undernutrition		COR (95%CI)	AOR (95% CI)	*P*-value
		Yes	No			
Household food security status	Secured	62	162	Ref.		
	Not secured	154	123	3.3 (2.3–4.7)	3.1 (2.1–4.6)	< 0.001
Dietary diversity score	Low	148	127	4.7 (2.6–8.4)	4.9 (2.6–9.2)	< 0.001
	Medium	53	97	2.2 (1.2–4.30)	2.3 (1.2–4.7)	0.016
	High	15	61	Ref.		
Latrine availability	Yes	99	172	Ref.		
	No	117	113	1.8 (1.3–2.6)	1.8 (1.2–2.6)	0.004
Resource decision making	Husband only	89	94	1.5 (1.0–2.4)	1.7 (1.1–2.6)	0.011
	Wife only	7	7	1.5 (0.5–4.4)	1.9 (0.6–6.0)	0.295
	Both equally	120	184	Ref.		

*COR* Crude odds ratio, *AOR* Adjusted odds ratio, *Ref* Reference category, *CI* Confidence interval

### Focus group discussion

Focus group discussion was held with 12 participants in each two FGD from different community groups such as pregnant mothers, husbands of pregnant mothers, mother in-low, and elders. Different barriers related to pregnant mother’s nutrition in the study area were explored during the focus group discussion session. Responses were coded and categorized by content with thematic analysis. These were: food restrictions, cultural believe related barriers, food production-related barriers, and food diversification practice-related barriers (Table 5).

**Table 5:** Food restrictions, cultural beliefs, food production, and food diversification-related barriers found from FGD in Konso district, southern Ethiopia, 2019

Categories	Responses
1. Food restrictions, cultural believe related barriers.	A 31 years old age pregnant mother said: *“… Culturally, pregnant women are not allowed to eat egg, butter, milk, other milk products, and avocado during pregnancy. The reason why it is not allowed to eat the list of food items above is, we believe when the pregnant mother ate these foods, the baby became very big and our fear is the mother will face difficulty in childbirth.”*
	Another 31 years pregnant woman said: *There is one quote related to using butter in a meal during pregnancy and this quote is used when people explaining something that is not relevant to be discussed or told before it is real-time or proud with something which is hope but not at hand*. This saying is: ***“****Why you drink butter before the fetus delivered from mother’s womb”*
	A 35 years old participant *“… The practice and attitude of some specific parts of food restriction is currently changing but commonly practiced in illiterate community and traditional believes followers.”*
	A 58 years mother in low responded for the time when some foods restricted during pregnancy as:- *“… It is from her early pregnancy or as she knows she is pregnant, mostly since the 4th month or second trimester of pregnancy. She also stated, the Cause for physical health deterioration in my opinion is; there is a food shortage in our area, but food sharing practice within the family in our community has its problem. The community gives priority to husbands and elders in food sharing and mothers who engaged in food sharing and small kids left with residual. Sometimes the remaining food might not adequately support mothers in their nutrition and mother will fall in food inadequate.”*
	A 38 years old pregnant woman’s husband said: “… *Off course, our area is drought-prone and frequently affected by drought, but besides that, some husbands have not given attention to their wives and family because our community is male dominant and males are the decision-makers for all family-related issues. on the other hand, males are mobile (moves from place to place for work-related purposes) and can have an opportunity to get a variety of foods but mothers and small kids are restricted to stay at home so that they lack the opportunity to get a variety of food.”*
2. Food production-related barriers	Agriculture and natural resource office head said: **“…** *The area is drought-prone frequently affected by drought. The farming is rain-dependent and the rainfall pattern is inerratic every year, the soil is seriously degraded and the landholding size is also very small which is 0.25–0.5 ha per household which cannot adequately be the source for family food”*
	Agriculture and natural resource department head said: “… *Food shortage is common in our district, currently the district is categorized as priority one district in early warning, four thousand six hundred twenty-seven people included in safety net program,31,570 people are exposed to emergence, almost one-third of the population is in food insecurity status.”*
3. Food diversification practice-related barriers	A community health worker *said: “… The health extension workers in the community are giving nutrition education in food diversification. However, nutrition counseling uptaking and implementation by our mothers is compromised, almost all households have chicken, producing egg and fruits grown in the garden but, most of them used to sell for household income rather than used for consumption. This indicates the poor nutritional status of pregnant women is not only associated with lack of food but also diversification of food is not commonly practiced”*
	A community health worker said: **“…** *The feeding practice in our community is a little bit different, the food content is not diversified, taking snacks and additional meal during pregnancy is not common and nutrition education activities at the community level are not strong besides to very low socio-economic status of the community”* *“… Family meal preparation trend is also totally changed in my observation, the meal for the family is only prepared once at night and the whole day people are depending on a locally prepared drink called Chaka including pregnant mothers itself.***”** community health workers *Again he said, “Our district is supported by therapeutic supplementary feeding program for pregnant women and lactating mothers and children under five years of age, but community-level health services such as screening mothers and children for their nutritional status is not strong.”*

## Discussion

The purpose of this study was to assess the prevalence and associated factors of undernutrition among pregnant women in Konso district, southern Ethiopia. This study found that four in every ten pregnant women were undernourished. Household food security status, dietary diversity, latrine availability, family resource decision-making were significant determinants of undernutrition. Food restriction practices, weak nutrition education and malnutrition screening program, the practice of depending on a local alcoholic drink called “Cheka”, drought, rain-dependent farming practices, and low socioeconomic status were identified barriers from qualitative data.

The current study found that the prevalence of undernutrition in pregnant women was 43.1% (95% CI: 38.8%-47.5). This finding was in agreement with the reported result from a rural community in southern Ethiopia which was 41.2% [22]. The result of this study was higher than those studies conducted in other areas of Ethiopia, 31.8% in Central Refit Valley [15], 9.2% in Wondogenet District southern Ethiopia [16], 21.8% in Silte zone southern Ethiopia [17], 19.8% in Dessie town, northeastern Ethiopia [14], 19.5% in Eastern Ethiopia [13], 24% in humanitarian setting in Ethiopia [18], 16.2% in Gondar hospital [20] and 14% in Gondar town [21]. The discrepancy might be due to differences in socio-demographic characteristics, geographical variation, cultural beliefs such as food taboos, poor nutritional intervention programs in the Konso district. Another possible reason may be due to a difference in the season of studies conducted.

The finding of this study was also higher than the report from Madagasgar, a systematic review in Africa, South Sudan, Kenya, China, India, Sri Lanka, Nigeria, 9, 18.9, 19.3, 23.5, 11.8, 5, 15, and 10%, respectively [11, 12, 24, 27, 41–44]. This variation might be due to differences in MUAC cut of value, and the socio-culture distinctions between Ethiopia and the other counties.

The household food security status was significantly associated with pregnant women undernutrition. The odds of undernutrition among pregnant women who had family food insecurity status were three times higher than with those women who had secured family food (AOR = 3.1; 95% CI: 2.1–4.6). This finding is similar to the previous study conducted in Gambella and Arbaminch Zuria Woreda Ethiopia [45, 46]. This could be possibly due to that family food shortage usually results in a lack of daily nutritional requirements and poor dietary intake leading to undernutrition of women. This finding was also consistent with the information given from one key informant: - “**…** *The area is drought-prone and frequently affected by drought. The farming is rain-dependent and the rainfall pattern is erratic every year, the soil is seriously degraded and the landholding size is also very small which is 0.25-0.5hector per household which cannot adequately be the source for family food”* Agriculture and natural resource office head.

Dietary diversity score was one of the factors significantly associated with undernutrition among pregnant mothers. The odds of undernutrition among pregnant women with low dietary diversity score were about five times higher when compared with those women with high dietary diversity score (AOR = 4.9; 95% CI: 2.6–9.2) and the odds of undernutrition among pregnant women with medium dietary diversity score were two times higher when compared with those women with high dietary diversity score (AOR = 2.3; 95% CI: 1.2–4.7). This result is in line with the study conducted in Gambella Ethiopia [45]. This might be because mothers who have a practice of food diversity will get the different nutrients from different diets and this might cause them to be well-nourished than those with less than average dietary diversity score.

The result was also consistent with data from focus group discussion; the traditional belief that restricts the consumption of some parts of foods with high proteins and energy-providing foods such as butter, milk, milk products, egg, and avocado during pregnancy. As one of the participants said:- *“… Culturally, pregnant women are not allowed to eat egg, buttermilk, other milk products, and Avocado during pregnancy. The reason why it is not allowed to eat the list of food items above is, we believe when the pregnant mother ate these foods, the baby became very big and our fear is the mother will face difficulty in childbirth.”* 31 years old FGD Participant*.* Therefore, this can also affect the dietary diversity of pregnant women and lead to the poor nutritional status of pregnant mothers.

Latrine availability at the household level was significantly associated with the undernutrition of pregnant women. The odds of undernutrition among pregnant women without a latrine in their house were 1.8 times higher when compared to the counterparts (AOR = 1.8; 95%CI: 1.2–2.6). This result is in line with the study conducted in Tanzania [47]. The possible explanation may be those pregnant women who have no latrine and using open field defecation have higher exposure to recurrent diarrheal disease and other infections these may lead to the poor nutritional status of pregnant mothers since infection and malnutrition have a direct relationship and it is an immediate cause for undernutrition.

The decision-making power on household assets had also a significant association with pregnant women undernutrition. The odds of undernutrition among pregnant women who had family resource decision-making by husband only were 1.7 times higher than those who made the decision both husband and wife (AOR = 1.7; 95%CI: 1.1–2.6). This finding is in line with findings from a qualitative study conducted in three food-insecure districts of Tigray Region, Northern Ethiopia [48]. This might be due to the reason that one part wife only or husband only decision in family resources may affect their communication and if one ignores the idea of others, this may cause the wives not to be supported by their husbands and this may also negatively affect their nutritional habit. In addition to that, the finding was consistent with qualitative data from focus group discussion:- *“… Some husbands have not given attention for their wives and family because our community is male dominant and males are the decision-makers for all family-related issues. On the other hand, males are mobile (moves from place to place for work-related purposes) and can have an opportunity to get a variety of foods but mothers and small kids are restricted to stay at home so that they lack un opportunity to get a variety of food.”* FGD participant.

### Limitations of the study

This study recognized the following limitations: this study used MUAC < 23 cm as the cut-off value for undernutrition in pregnant women. Currently, there is no consensus on how to identify pregnant as acutely malnourished but according to Ververs MT et al., MUAC was identified as the preferential indicator of choice this is because of its relatively strong association with low birth weight, narrow range of cut-off values, simplicity of measurement and it does not require prior knowledge of gestational age and a conservative cut-off < 23 cm was recommended in African contexts to include most pregnant women of low birth weight for their infants [34]. Some self-reported variables like household food security status and decision-making power on the household assets may be affected by social desirability bias and it was reduced through detailed clarification of the objective before entering into individual interviews. In dietary diversity assessment since 24-h recall data collection (food listing method) used, thorough interview process recall bias is expected (reduced by probing).

## Conclusions

The prevalence of undernutrition among pregnant women was found to be higher than previously reported findings in Ethiopia and other countries. Food security, dietary diversity, latrine availability, family resource decision making, food restriction, weak nutrition education, and malnutrition screening program, the practice of depending on a local alcoholic drink called “Cheka”, drought, poor hygiene and sanitation coverage, traditional way of farming and low socio-economic status were identified factors. Hence, interventions targeting maternal nutrition education, personal hygiene, and sanitation, encouraging irrigation through working with the agricultural sector to change the traditional way of farming practices and the economic status of the community are recommended.

## Data Availability

All relevant data are available from the corresponding author upon reasonable request.

## Abbreviations

ANC: Antenatal care
DD: Dietary diversity
DDS: Dietary diversity score
EDHS: Ethiopian Demographic and Health Survey
FAO: Food and Agriculture Organization
FS: Food security
IUGR: Intrauterine Growth Retardation
IFAD: International fund for agriculture and development
MUAC: Mid-Upper Arm Circumference
SPSS: Statistical Package for Social Science
WFP: World Food Program
WHO: World Health Organization

## References

[1] Dalky H, Qandil A, Alqawasmi A (2018). Factors associated with Undernutrition among pregnant and lactating Syrian refugee women in Jordan. Global J Health Sci.

[2] Prajakta Ganesh Joshi G, Jain S, Dubey V. Nutritional status of pregnant women reporting at rural health training centre international. J Reprod Contraception Obstet Gynecol. 2017;6(9).

[3] UNICEF (2018). The lancet series on maternal and child undernutrition.

[4] Skinner A-L (2012). Pregnancy outcome in south Asian women: factors affecting diet and nutrition: University of Central Lancashire.

[5] Daba G, Beyene F, Fekadu H, Garoma W (2013). Assessment of knowledge of pregnant mothers on maternal nutrition and associated factors in Guto Gida Woreda, east Wollega zone, Ethiopia. J Nutr Food Sci.

[6] Martin-Gronert MS, Ozanne SE (2006). Maternal nutrition during pregnancy and health of the offspring. Biochem Soc Trans.

[7] Imdad A, Bhutta ZA (2012). Maternal nutrition and birth outcomes: effect of balanced protein-energy supplementation. Pediatr Perinat Epidemiol.

[8] Bagriansky J, Champa N, Pak K, Whitney S, Laillou A (2014). The economic consequences of malnutrition in Cambodia, more than 400 million US dollars lost annually. Asia Pac J Clin Nutr.

[9] Lewit EM, Baker LS, Corman H, Shiono PH (1995). The direct cost of low birth weight. Futur Child.

[10] Liu FLZY, Parés GV, Reidy KC, Zhao WZ, Zhao A, Chen C, et al. Nutrient intakes of pregnant women and their associated factors in eight cities of China: a cross-sectional study. Chin Med J. 2015;128(13). 10.4103/0366-6999.159354 PMC4733713 26112720

[11] Adikari A, Sivakanesan R, Wijesinghe D, Liyanage C (2016). Assessment of nutritional status of pregnant women in a rural area in Sri Lanka. Assessment of nutritional status of pregnant women in a rural area in Sri Lanka.

[12] EA. U. Nutritional practices and taboos among pregnant women attending antenatal care at the general hospital in Kano, Northwest Nigeria. . Ann Med Health Sci Res 2016;6(2):109–114, DOI: 10.4103/2141-9248.181846. 10.4103/2141-9248.181846 PMC4866363 27213094

[13] Kedir HBY, Worku A (2016). Magnitude and determinants of malnutrition among pregnant women in eastern E Ethiopia: evidence from rural, community-based setting. Matern Child Nutr.

[14] Diddana TZ (2019). Factors associated with dietary practice and nutritional status of pregnant women in Dessie town, northeastern Ethiopia: a community-based cross-sectional study. BMC Pregnancy Childbirth.

[15] Mariyam AFDB (2018). Epidemiology of malnutrition among pregnant women and associated factors in central refit valley of Ethiopia, 2016. J Nutr Disord Ther.

[16] Desalegn KPS, Debebe M (2015). Dietary practices and associated factors among pregnant women in Wondo genet district, southern Ethiopia: a cross-section study. J Pharm Sci Innov.

[17] Muze MYM, Kedir S, Mustafa A (2020). Prevalence and associated factors of undernutrition among pregnant women visiting ANC clinics in Silte zone, Southern Ethiopia. BMC Pregnancy Childbirth.

[18] Gebre BBS, Taddese Z, Legesse T, Letebo M (2018). Determinants of malnutrition among pregnant and lactating women under humanitarian setting in Ethiopia. BMC Nutr.

[19] Belete YNB, Firehiwot M. Undernutrition and associated factors among adolescent pregnant women in Shashemene, west Arsi zone, Ethiopia: a community-based study. J Nutr Food Sci. 2016.

[20] Kumera GGD, Alebel A, Feyera F, Eshetie S (2018). Undernutrition and its association with socio-demographic, anemia and intestinal parasitic infection among pregnant women attending antenatal care at the University of Gondar Hospital, Northwest Ethiopia. Matern Health Neonatol Perinatol.

[21] Dadi AFDH. Undernutrition and its associated factors among pregnant mothers in Gondar town, Northwest Ethiopia. PloS One. 2019;14(4). 10.1371/journal.pone.0215305 PMC6476509 31009475

[22] Zewdie S, Fage SG, Tura AK, Weldegebreal F (2021). Undernutrition among pregnant women in rural communities in southern Ethiopia. Int J Womens Health.

[23] I H. Reduction of food intake during pregnancy in rural South India. Tropical Med Int Health 1996; 1(3):399–405, DOI: 10.1046/j.1365-3156.1996.d01-53.x. 10.1046/j.1365-3156.1996.d01-53.x 8673846

[24] Abasizadeh SHZ, Deres F (2016). Prevalence of malnutrition during pregnancy and associated factors in women of Ardal County in 2012–2013. Int J Epidemiol Res.

[25] Chinnakali PUR, Shokeen D, Singh K, Kaur M, Singh AK, Goswami A, Yadav K, Pandav CS (2014). Prevalence of household-level food insecurity and its determinants in an urban resettlement colony in north India. J Health Popul Nutr.

[26] Zhou DST, Ali S, Ahmad W, Din IU, Ilyas A (2019). Factors affecting household food security in the rural northern hinterland of Pakistan. J Saudi Soc Agric Sci.

[27] Kiboi WKJ, Chege P. Dietary diversity, nutrient intake and nutritional status among pregnant women in Laikipia County, Kenya. Int J Health Sci Res. 2016:378–9.

[28] Lee SETS, Merialdi M, Caulfield LE (2013). Dietary intakes of women during pregnancy in low-and middle-income countries. Public Health Nutr.

[29] Ayensu J, Annan R, Lutterodt H, Edusei A, Peng LS. Prevalence of anemia and low intake of dietary nutrients in pregnant women living in rural and urban areas in the Ashanti region of Ghana. PLoS One. 2020;15(1). 10.1371/journal.pone.0226026 PMC6980408 31978048

[30] Daniel SPG, Gnanaraj S, Sharmine E (2016). Effect of nutrition education among pregnant women with low body mass index: a community-based intervention. Int J Community Med Public Health.

[31] Acharya SRBJ, Timilsina DP (2017). Factors associated with nutritional status of women of reproductive age group in rural, Nepal. Asian Pac J Health Sci.

[32] F. H (2016). Maternal factors, early pregnancy anthropometry, and gestational weight gain: a population-based study in Matlab, Bangladesh.

[33] Report K (2018). Pregnant women undernutrition.

[34] Ververs MTAA, Sackl A, Staderini N, Captier V. Which anthropometric indicators identify a pregnant woman as acutely malnourished and predict adverse birth outcomes in the humanitarian context? PLoS Curr. 2013;7(5). 10.1371/currents.dis.54a8b618c1bc031ea140e3f2934599c8 PMC3682760 23787989

[35] TB GK, Marie C (2013). Guidelines for measuring household and individual dietary diversity.

[36] EDHS (2016). Central statistical agency [Ethiopia] and ORC Macro. Ethiopia Demographic and Health Survey (EDHS).

[37] Tang AMCM, Dong K, Terrin N, Edmonds A, Assefa N, Maalouf-Manasseh Z. Determining a global mid-upper arm circumference cutoff to assess malnutrition in pregnant women: Food and Nutrition Technical Assistance; 2016.

[38] F. F (2016). Minimum dietary diversity for women: a guide for measurement. Rome: FAO. 2016;82./ FAO, Minimum Dietary Diversity for Women; A Guidelines to Measurement, Food and Agriculture Organization of the United Nations.

[39] Coates JSA, Bilinsky P (2007). Household food insecurity access scale (HFIAS) for measurement of food access: indicator guide: version 3.

[40] Girard AWSJ, McAuliffe C, Olude O (2012). The effects of household food production strategies on the health and nutrition outcomes of women and young children: a systematic review. Paediatr Perinat Epidemiol.

[41] Ravaoarisoa LRL, Rakotonirina J, Rakotomanga JD, Donnen P, Dramaix MW (2018). Socioeconomic determinants of malnutrition among mothers in the Amoron’i Mania region of Madagascar: a cross-sectional study. BMC Nutr.

[42] DA Desyibelew HD. Burden and determinants of malnutrition among pregnant women in Africa: a systematic review and meta-analysis. PLoS One. 2019;6, 14(9). 10.1371/journal.pone.0221712 PMC6730925 31490956

[43] Alemayehu AGL, Yemane T, Asres Y. Prevalence, severity, and determinant factors of anemia among pregnant women in South Sudanese refugees, Pugnido, Western Ethiopia. Anemia. 2016:2016. 10.1155/2016/9817358 PMC5183745 28058116

[44] Gao HSC, Scherbaum V, Biesalski HK, Wang Q, Hormann E, Bellows AC (2013). Dietary intake and food habits of pregnant women residing in urban and rural areas of Deyang City, Sichuan Province, China. Nutrients..

[45] Nigatu MGT, Gemeda DH (2018). Household food insecurity, low dietary diversity, and early marriage were predictors for Undernutrition among pregnant women residing in Gambella, Ethiopia. Adv Public Health.

[46] Tikuye HHGS, Mesfin A, Whiting S. Prevalence and factors associated with undernutrition among exclusively breastfeeding women in Arba Minch Zuria District, Southern Ethiopia: a cross-sectional community-based study. Ethiop J Health Sci. 2019;29(1). 10.4314/ejhs.v29i1.13 PMC6341443 30700959

[47] Mtumwa AHPE, Vuoi SA (2016). Determinants of undernutrition among women of reproductive age in Tanzania mainland. South Afr J Clin Nutr.

[48] Ayele E, Gebreayezgi G, Mariye T, Bahrey D, Aregawi G, Kidanemariam G (2020). Prevalence of Undernutrition and associated factors among pregnant women in a public general hospital, Tigray, northern Ethiopia: a cross-sectional study design. J Nutr Metab.

